# Kinetic and structural characterization of amyloid‐β peptide hydrolysis by human angiotensin‐1‐converting enzyme

**DOI:** 10.1111/febs.13647

**Published:** 2016-02-09

**Authors:** Kate M. Larmuth, Geoffrey Masuyer, Ross G. Douglas, Sylva L. Schwager, K. Ravi Acharya, Edward D. Sturrock

**Affiliations:** ^1^ Department of Integrative Biomedical SciencesInstitute of Infectious Disease and Molecular MedicineUniversity of Cape TownSouth Africa; ^2^ Department of Biology and BiochemistryUniversity of BathUK; ^3^ Present address: Department of Biochemistry and BiophysicsArrhenius Laboratories for Natural SciencesStockholm University10691StockholmSweden; ^4^ Present address: Integrative ParasitologyDepartment of Infectious DiseasesUniversity of Heidelberg Medical SchoolIm Neuenheimer Feld 32469120HeidelbergGermany

**Keywords:** Alzheimer disease, cooperativity, crystallography, enzyme kinetics, metalloprotease

## Abstract

Angiotensin‐1‐converting enzyme (ACE), a zinc metallopeptidase, consists of two homologous catalytic domains (N and C) with different substrate specificities. Here we report kinetic parameters of five different forms of human ACE with various amyloid beta (Aβ) substrates together with high resolution crystal structures of the N‐domain in complex with Aβ fragments. For the physiological Aβ(1–16) peptide, a novel ACE cleavage site was found at His14‐Gln15. Furthermore, Aβ(1–16) was preferentially cleaved by the individual N‐domain; however, the presence of an inactive C‐domain in full‐length somatic ACE (sACE) greatly reduced enzyme activity and affected apparent selectivity. Two fluorogenic substrates, Aβ(4–10)Q and Aβ(4–10)Y, underwent endoproteolytic cleavage at the Asp7‐Ser8 bond with all ACE constructs showing greater catalytic efficiency for Aβ(4–10)Y. Surprisingly, in contrast to Aβ(1–16) and Aβ(4–10)Q, sACE showed positive domain cooperativity and the double C‐domain (CC‐sACE) construct no cooperativity towards Aβ(4–10)Y. The structures of the Aβ peptide–ACE complexes revealed a common mode of peptide binding for both domains which principally targets the C‐terminal P2′ position to the S2′ pocket and recognizes the main chain of the P1′ peptide. It is likely that N‐domain selectivity for the amyloid peptide is conferred through the N‐domain specific S2′ residue Thr358. Additionally, the N‐domain can accommodate larger substrates through movement of the N‐terminal helices, as suggested by the disorder of the hinge region in the crystal structures. Our findings are important for the design of domain selective inhibitors as the differences in domain selectivity are more pronounced with the truncated domains compared to the more physiological full‐length forms.

**Database:**

The atomic coordinates and structure factors for N‐domain ACE with Aβ peptides 4–10 (5AM8), 10–16 (5AM9), 1–16 (5AMA), 35–42 (5AMB) and (4–10)Y (5AMC) complexes have been deposited in the Protein Data Bank, Research Collaboratory for Structural Bioinformatics, Rutgers University, New Brunswick, NJ, USA (http://www.rcsb.org/).

**Abbreviations**
Aβ: amyloid beta
ACE: angiotensin‐1‐converting enzyme
ACN: acetonitrile
AD: Alzheimer's disease
CC‐sACE: double C‐domain of sACE
Cdom: soluble form of the C‐domain of sACE
CHO: Chinese hamster ovary cells
EDDnp: ethylenediamine 2,4‐dinitrophenyl
Ndom389: minimally glycosylated Ndom
Ndom: soluble form of the N‐domain of sACE
NT: 3‐nitrotyrosine
RAAS: renin‐angiotensin‐aldosterone system
sACE: somatic ACE
tACE: human testis ACE
TFA: trifluoroacetic acid

## Introduction

Angiotensin‐1‐converting enzyme (ACE, EC 3.4.15.1) is a zinc peptidase that plays a pivotal role in the renin‐angiotensin‐aldosterone system (RAAS) converting angiotensin I to the vasoactive peptide hormone angiotensin II 1, 2. ACE also cleaves the vasodilatory peptide bradykinin further enhancing the blood pressure response 3. Interestingly, in humans, somatic ACE (sACE) consists of two catalytically active domains 4, 5 (referred to as the N‐ and C‐domains) that, while very similar in sequence and structural topology, display differences in substrate processing abilities 6, 7, 8. In addition to the cleavage of vasopeptides, ACE is able to cleave a variety of peptides that are unrelated to its blood pressure role. While these are perhaps not the most well‐known ACE substrates, many have important physiological roles. For instance, there exists an independent brain RAAS that extends its role beyond fluid and blood pressure homeostasis into areas such as sexual behaviour, cerebroprotection, diabetes, psychological disorders and many neurodegenerative diseases including Alzheimer's disease (AD) 9, 10, 11, 12, 13, 14. ACE's specific role in AD is not entirely clear and is somewhat controversial 15, 16, 17, 18, 19, 20. However, it has been shown that ACE hydrolyses the amyloid beta (Aβ) peptide, the putative causative agent of AD 20, 21, 22, 23, 24, 25, 26.

The Aβ peptide is present in soluble and aggregated extracellular masses in the vasculature and neuronal tissue of AD patients 27, 28. Aβ is formed from the alternate processing of the transmembrane amyloid precursor protein via sequential cleavage by the β‐ and γ‐secretases 28, 29, 30, 31. The most well‐known toxic form of Aβ is 42 residues long, although Aβ also occurs in many truncated variants and in more soluble forms 32. These more soluble oligomers and protofibrils are strongly correlated with increased toxicity compared to the more structured fibrils 33, 34, 35, 36. The Aβ(1–16) amyloid variant is formed via both amyloidogenic and non‐amyloidogenic secretion pathways; however, it has a controversial role in AD being indicated as both inert and cytotoxic 37, 38, 39. The cytotoxic properties are linked to the metal binding domain of Aβ(1–16), known for its role in producing detrimental reactive oxygen species and aiding oligomerization on its own and within the full‐length Aβ(1–42) 38, 39, 40, 41. Moreover, Aβ(1–16) formation has been found to be upregulated in AD cerebrospinal fluid 42, 43. There is conflicting evidence regarding N‐ and C‐domain selectivity of Aβ cleavage by ACE and the enzyme's P1 and P1′ preference. The general trend of results indicates that the N‐domain alone is more effective at Aβ cleavage, making it more selective, compared to the C–domain 21, 25, 26. However, in sACE the C‐domain appears to have equivalent selectivity to the N‐domain 22, 24. The absolute selectivity and cooperative interactions between the two domains of ACE and Aβ remain uncharacterized. The N‐ and C‐domains of ACE have been shown to display negative cooperativity in substrate hydrolysis 44, 45, 46. While this is observed with many synthetic and naturally occurring peptides, not all substrates displayed such an effect 46. Therefore we rigorously investigated the kinetics of Aβ peptide metabolism by different full‐length and single domain ACE constructs under defined experimental conditions. We analysed the kinetics of hydrolysis of Aβ(1–16) and two fluorogenic peptides of the N‐terminal region of Aβ by the human sACE containing both active sites, full‐length and single domain ACE containing one active site, and ACE with two tandem C‐domains to provide a biochemical basis for (a) the potential role of each active site and (b) possible synergistic effects of the two domains. Furthermore we interrogated the cleavage site specificity of the ACE enzymes and the molecular basis of the Aβ peptide binding to the N‐domain using high resolution crystal structures of the enzyme in complex with five soluble Aβ fragments.

## Results

### Purification of ACE variants

**Figure 1:**
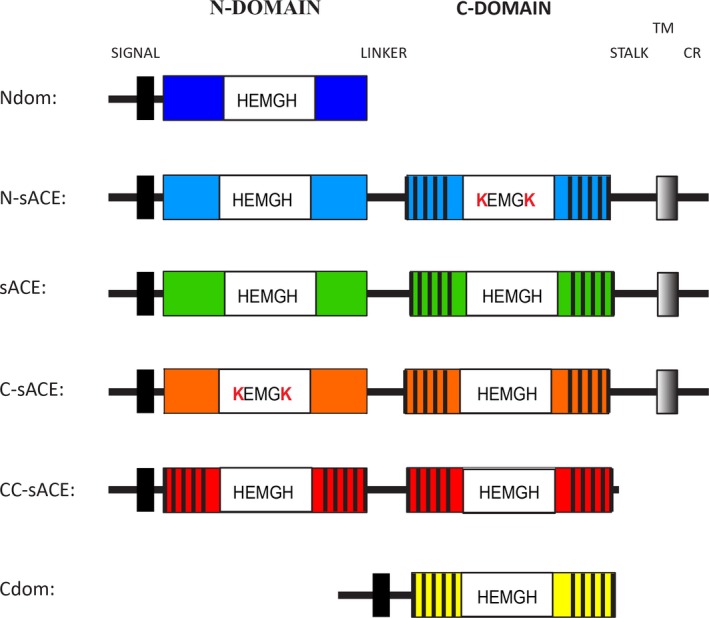
Schematic of the ACE constructs used. The wild‐type ACE (sACE) contains a signal peptide (black box), the two homologous ectodomains (C‐domain is indicated with black stripes) with the indicated active site residues necessary for the coordination of Zn, a stalk region, transmembrane domain (grey shaded box) and cytoplasmic region (CR). The truncated Ndom (dark blue) contains the signal peptide and the N‐domain. The truncated Cdom (yellow) is essentially the same as the C‐domain in wild‐type sACE only it lacks the transmembrane region and has the added signal peptide. The domain inactivated mutants are identical to sACE except for the mutation of the catalytic His residues to Lys (white boxes) in the N‐domain (N‐sACE) (blue) and C‐domain (C‐sACE) (orange). The CC‐sACE (red) construct is a fusion construct of two tandem copies of C‐domain.

Six human ACE variants were used to examine the molecular mechanism of domain selectivity and interdomain cooperativity towards Aβ substrates (Fig. 1). Human wild‐type sACE consists of a cytoplasmic tail, transmembrane domain and catalytically active N‐ and C‐domains. Full‐length domain knockout variants of sACE have had the critical zinc coordinating His residues converted to Lys residues, in the C‐domain (N‐sACE) and N‐domain (C‐sACE), inactivating each domain respectively. The full‐length double C‐domain construct consists of two C‐domains in a sACE form (CC‐sACE). The truncated, single domain enzymes are in essence the C‐ and N‐domains of sACE in soluble form (referred to as Cdom and Ndom). For all crystallization experiments a minimally glycosylated form of Ndom (Ndom389) 47 was used as glycosylation hinders the crystallization of ACE. The Ndom389 construct has only three intact glycosylation sites, sites 3, 8 and 9. This is the minimal site occupancy of the Ndom required to maintain the structural and functional integrity of the enzyme.

**Figure 2:**
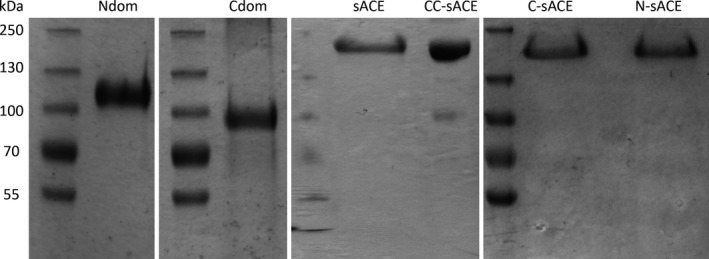
SDS/PAGE of the purified ACE variants. The affinity‐purified preparations of recombinant human ACE (approximately 10 μg of protein each) were analysed by SDS/PAGE as described in Experimental procedures. Molecular mass markers (kDa) are indicated on the left.

The human sACE, C‐sACE, N‐sACE, Ndom (including Ndom389), Cdom and CC‐sACE variants (Fig. 1) were expressed in mammalian cells and then purified using lisinopril‐sepharose affinity chromatography. The sACE, C‐sACE and N‐sACE constructs are expressed as membrane‐bound proteins and undergo poor ectodomain shedding 48, 49. Hence, these constructs were purified directly from cell lysate. CC‐sACE is also membrane bound but is shed much more efficiently and so was purified from the culture medium. All ACE variants were purified to apparent homogeneity as assessed by SDS/PAGE (Fig. 2). The sACE proteins migrated at approximately 170 kDa, while the recombinant Ndom and Cdom migrated with an apparent molecular mass of 100 kDa and 78 kDa, respectively.

### Kinetics of the hydrolysis of β‐amyloid peptides by ACE

We determined the kinetic constants for the hydrolysis of various Aβ peptides by two separate domains of ACE and full‐length ACE variants in order to further delineate domain selectivity and probe cooperative effects.

**Figure 3:**
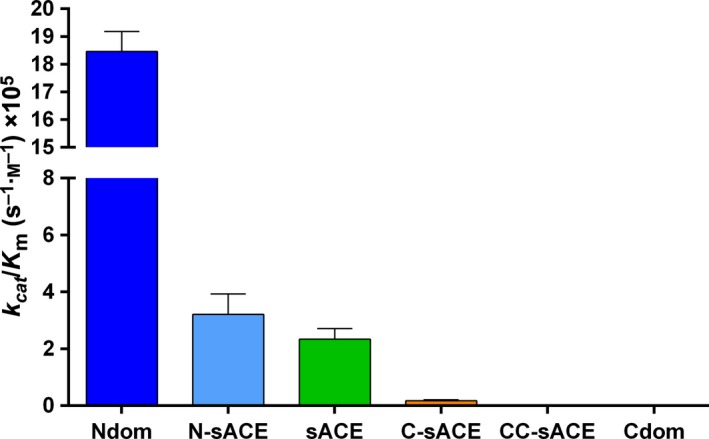
Graphical representation of the overall kinetic efficiency of Aβ(1–16). The data indicate hydrolysis of Aβ(4–10)Q by truncated, full‐length and wild‐type ACE constructs (error bars indicate the fractional error of the ratio; *n* = 3; colour coding as in Fig. 1).

Aβ(1–16) was cleaved most efficiently by the truncated Ndom (*k*_cat_/*K*_m_ 18.46 × 10^5^ m ^−1^·s^−1^), but was not hydrolysed by the truncated Cdom even after prolonged incubation times (Fig. 3, Table 1). A similar trend was found with the full‐length sACE constructs although the *k*_cat_/*K*_m_ for N‐sACE was 6‐fold lower (*P* < 0.05) than that for Ndom. The varying efficiencies between N‐sACE and C‐sACE are due to a difference in *k*_cat_. The CC‐sACE construct was investigated to see if there was any structural or physical effect on the C‐domain of sACE through the presence of another protein other than the N‐domain. Similarly to the Cdom, CC‐sACE processed Aβ(1–16) poorly compared to C‐sACE. Although CC‐sACE did process Aβ(1–16) the rate was too slow to generate accurate kinetic constants (Fig. 3, Table 1).

**Table 1:** Kinetic parameters of Aβ(1–16) (H‐DAEFRHDSGYEVHHQK‐OH) hydrolysis by different ACE variants/molecules. Error is represented as standard error of the mean (±SEM) barring the *k*_cat_/*K*_m_ values where the error represents the fractional error of the ratio; *n* = 3. ND, not determined

Enzyme	*k*_cat_/*K*_m_ (m ^−1^·s^−1^ × 10^5^)	*k*_cat_ (s^−1^)	*K*_m_ (μm)	*V*_max_	*E* (pmol)
Ndom	18.46 ± 0.72	39.30 ± 0.47	21.35 ± 0.21	0.49 ± 0.02	0.013
N‐sACE	3.21 ± 0.72	11.03 ± 1.62	34.20 ± 5.90	0.55 ± 0.08	0.050
sACE	2.36 ± 0.37	8.37 ± 0.90	35.20 ± 4.03	1.00 ± 0.19	0.120
C‐sACE	0.17 ± 0.04	0.67 ± 0.08	42.36 ± 9.57	1.15 ± 0.14	1.750
CC‐sACE	ND	ND	ND	ND	ND
Cdom	ND	ND	ND	ND	ND

**Figure 4:**
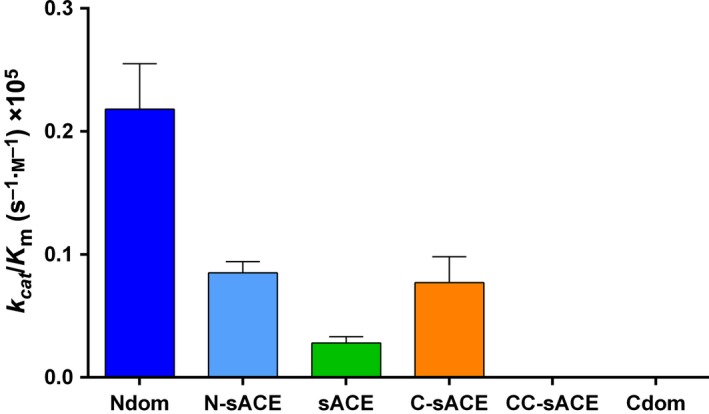
Graphical representation of the overall kinetic efficiency of Aβ(4–10)Q. The data indicate hydrolysis of Aβ(4–10)Q by truncated, full‐length and wild‐type ACE constructs (error bars indicate the fractional error of the ratio; *n* = 3; colour coding as in Fig. 1).

Fluorogenic peptides are widely used in protease assays. To validate the use of a fluorogenic Aβ peptide, we examined the ability of different ACE constructs to hydrolyse fluorogenic Aβ(4–10) peptides. The Aβ(4–10)Q peptide was hydrolysed very slowly by both Ndom and N‐sACE (*k*_cat_/*K*_m_ 0.22 × 10^5^ and 0.09 × 10^5^ m ^−1^·s^−1^, respectively) (Table2). However, the trend of the full‐length and truncated ACE constructs was similar to the physiological Aβ(1–16), being more selective towards the truncated Ndom. CC‐sACE displayed almost no hydrolysis towards the Aβ(4–10)Q substrate, similar to Aβ(1–16) (Fig. 4, Table 2).

**Table 2:** Kinetic parameters of Aβ(4–10)Q (Abz‐AβFRHDSG(Q)‐EDDnp) hydrolysis by different ACE variants/molecules. Error is represented as standard error of the mean (±SEM) barring the *k*_cat_/*K*_m_ values where the error represents the fractional error of the ratio; *n* = 3. ND, not determined

Enzyme	*k*_cat_/*K*_m_ (m ^−1^·s^−1^ × 10^5^)	*k*_cat_ (s^−1^)	*K*_m_ (μm)	*V*_max_	*E* (pmol)
Ndom	0.22 ± 0.04	0.48 ± 0.04	22.20 ± 3.23	0.29 ± 0.02	0.60
N‐sACE	0.09 ± 0.01	0.36 ± 0.02	42.43 ± 3.92	3.23 ± 0.20	9.00
sACE	0.03 ± 0.01	0.06 ± 0.01	22.90 ± 3.45	0.09 ± 0.01	1.50
C‐sACE	0.08 ± 0.02	0.08 ± 0.01	11.50 ± 3.03	0.12 ± 0.01	1.50
CC‐sACE	ND	ND	ND	ND	ND
Cdom	ND	ND	ND	ND	ND

**Figure 5:**
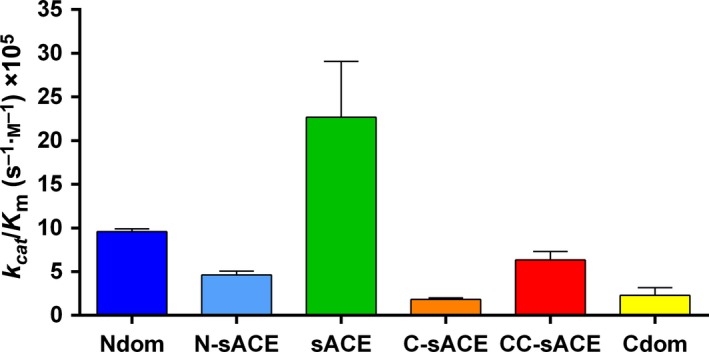
Graphical representation of the overall kinetic efficiency of Aβ(4–10)Y. Bar graphs represent the hydrolysis of Aβ(4–10)Y by truncated, full‐length and wild‐type ACE constructs (error bars indicate the fractional error of the ratio; *n* = 3; colour coding as in Fig. 1).

The Aβ(4–10)Y fluorogenic peptide was cleaved with half the catalytic efficiency to Aβ(1–16) by Ndom (9.57 × 10^5^ versus 18.46 × 10^5^ m ^−1^·s^−1^) (Fig. 5, Table 3). Contrary to the results of Aβ(1–16), sACE hydrolysed Aβ(4–10)Y more efficiently (2‐fold greater, *P* < 0.05) than Ndom; the increase in sACE's efficiency is due to the larger Ndom *K*_m_ as their turnover rates are equivalent. Notably there was a gain in C‐domain activity towards Aβ(4–10)Y. This is evident in the comparable efficiencies between Cdom, C‐sACE, N‐sACE and CC‐sACE. The C‐sACE *K*_m_, however, is 2‐fold (*P* < 0.05) larger than its active N‐domain counterpart accounting for C‐sACE's slightly lesser efficiency as the *k*_cat_ values are alike. Interestingly, the CC‐sACE construct cleaved the Aβ(4–10)Y with a higher *k*_cat_/*K*_m_ than N‐sACE but lower than the truncated Ndom. The overall turnover rate for CC‐sACE was much greater than either Cdom or C‐sACE (1.5‐fold increase over both, *P* < 0.05) towards Aβ(4–10)Y.

**Table 3:** Kinetic parameters of Aβ(4–10)Y (Abz‐FRHDSG) hydrolysis by different ACE variants/molecules. Error is represented as standard error of the mean (±SEM) barring the *k*_cat_/*K*_m_ values where the error represents the fractional error of the ratio; *n* = 3

Enzyme	*k*_cat_/*K*_m_ (m ^−1^·s^−1^ × 10^5^)	*k*_cat_ (s^−1^)	*K*_m_ (μm)	*V*_max_	*E* (pmol)
Ndom	9.57 ± 0.33	20.30 ± 0.62	21.20 ± 0.31	4.62 ± 0.14	0.228
N‐sACE	4.61 ± 0.46	3.87 ± 0.09	8.56 ± 0.83	0.44 ± 0.01	0.113
sACE	22.67 ± 6.40	28.70 ± 5.81	12.60 ± 2.48	0.90 ± 0.18	0.031
C‐sACE	1.83 ± 0.18	6.95 ± 1.39	38.10 ± 0.10	1.57 ± 0.09	0.226
CC‐sACE	6.33 ± 0.98	12.70 ± 1.16	20.30 ± 2.55	0.90 ± 0.08	0.071
Cdom	2.30 ± 0.87	5.33 ± 0.76	27.23 ± 9.52	4.45 ± 0.63	0.835

### Cleavage‐site analysis of β‐amyloid peptides

To determine the bond at which the Aβ peptides were cleaved by the different ACE variants, each substrate was incubated under the stated assay conditions and the peptide products were purified for MALDI‐TOF MS analysis. For the Aβ(4–10)Q and Aβ(4–10)Y, peptides with *m*/*z* = 694.3 were identified (Table4). This corresponds to the N‐terminal *o*‐aminobenzoyl (Abz)‐FRHD peptide. Thus, both Aβ(4–10) peptides were endoproteolytically cleaved at the Asp7‐Ser8 bond. The Cdom and CC‐sACE cleavage sites were not analysed as there was negligible hydrolysis of the Aβ peptides by these ACE variants under kinetic assay conditions. Hydrolysis of Aβ(1–16) by all the ACE variants except Cdom yielded peptides with *m*/*z* ratios of 1698.6 (Ndom), 1698.7 (N‐sACE, C‐sACE, CC‐sACE) and 1698.8 (sACE) within kinetic assay parameters. These peptides correspond to the N terminus Aβ(1–14) (DAEFRHDSGYEVHH) with a calculated mass of 1698.7 (Table 4). This indicates that Aβ(1–16) is cleaved at a different bond, namely His14‐Gln15. To determine if the Aβ(1–16) is further degraded over time by all forms of ACE, 36.5 μm of Aβ(1–16) was incubated for 24 h (Table 5). Interestingly, the mass of the initial cleavage intermediate Aβ(1–14), 1698.7 *m*/*z*, was present across all ACE constructs except for N‐sACE. However, the Aβ(1–12) (DAEFRHDSGYEV) peptide was only generated by the sACE mutants (expected *m*/*z* of 1325.6). The corresponding C‐terminal peptide, Aβ(12–16), with an expected *m*/*z* of 648.3 was detected in both Cdom and Ndom digests as well as in sACE and C‐sACE, but not N‐sACE or CC‐sACE. The Aβ(1–12) product is probably hydrolysed faster by the Cdom, Ndom and wild‐type sACE. Overall, there appears to be no absolute domain preference for any specific P1 and P1′ residues, similar to the hydrolysis for 15 min. The Aβ(1–7) cleavage site is also found across constructs barring N‐sACE and CC‐sACE. These results indicate that the hydrolysis of Aβ(1–16) by both domains of ACE is not limited or specific, and that under certain conditions the C‐domain does hydrolyse Aβ peptides.

**Table 4:** Aβ cleavage products produced from ACE hydrolysis. Observed [M + H]^+^ ions of the peptide products generated by endoprotease and exoprotease action of the various ACE constructs on the Aβ(1–16), Aβ(4–10)Q and Aβ(4–10)Y substrates. ND, not determined

	Peptide residues	Calculated *m*/*z*	Observed *m*/*z*					
			Ndom	N‐sACE	sACE	C‐sACE	CC‐sACE	Cdom
Aβ(1–16)								
Substrate	DAEFRHDSGYEVHHQK	1954.9	1954.8	1954.8	1954.8	1954.8	1954.8	1954.8
Product	DAEFRHDSGYEVHH	1698.7	1698.6	1698.7	1698.8	1698.7	1698.7	ND
Aβ(4–10)Q								
Substrate	Abz‐FRHDSG(Q)‐EDDnp	1191.5	1191.5	1191.5	1191.5	1191.5	ND	1191.7
Product	Abz‐FRHD	694.3	694.3	694.3	694.3	694.3	ND	ND
Aβ(4–10)Y								
Substrate	Abz‐FRHDSG‐(NT)	1064.4	1064.4	1064.4	1064.4	1064.4	ND	ND
Product	Abz‐FRHD	694.3	694.3	694.3	693.3	694.3	ND	ND

**Table 5:** Aβ cleavage products from prolonged ACE hydrolysis. The observed cleavage product [M + H]^+^ ions generated by the various ACE constructs on the Aβ(1–16) substrate over a 24‐h period. ND, not determined

Amyloid peptide	Peptide residues	Calculated *m*/*z*	Observed *m*/*z*					
			Ndom	N‐sACE	sACE	C‐sACE	CC‐sACE	Cdom
Aβ(1–16)	DAEFRHDSGYEVHHQK	1954.8	1954.8	1954.8	1954.8	1954.8	1954.8	1954.8
N‐terminal products								
Aβ(1–14)	DAEFRHDSGYEVHH	1698.7	1698.7	ND	1698.7	1698.7	1698.6	1698.7
Aβ(1–12)	DAEFRHDSGYEV	1424.6	1424.6	1424.6	1424.6	1424.6	1424.6	1424.6
Aβ(1–11)	DAEFRHDSGYE	1325.5	ND	1324.5	ND	1325.5	1325.5	ND
Aβ(1–7)	DAEFRHD	889.3	889.3	ND	889.3	889.3	ND	889.3
Aβ(1–6)	DAEFRH	774.4	ND	ND	ND	774.4	ND	ND
Aβ(1–5)	DAEFR	637.3	637.3	ND	637.3	637.3	637.3	637.3
C‐terminal products								
Aβ(2–16)	AEFRHDSGYEVHHQK	1839.8	ND	ND	ND	ND	ND	1839.8
Aβ(4–16)	FRHDSGYEVHHQK	1639.7	1639.7	ND	ND	ND	ND	ND
Aβ(12–16)	VHHQK	648.3	648.3	ND	648.3	648.3	ND	648.3

### Crystal structures of N‐domain in complex with β‐amyloid peptides

Crystallization trials were performed to analyse the molecular interactions of Ndom389 with Aβ. While Ndom389 co‐crystals with Aβ(1–42) could not be obtained, trials with shorter Aβ fragments, namely Aβ(4–10), Aβ(10–16), Aβ(1–16), Aβ(35–42) and Aβ(4–10)Y, were successful in solving high resolution (1.5–1.9 Å) structures of the complexes (Table 6).

**Table 6:** Crystallographic statistics of the structures of Ndom389 in complex with Aβ fragments. *R*_merge_ = ΣΣ_*i*_|*I*_*h*_ − *I*_*hi*_|/ΣΣ_*i*_ *I*_*h*_, where *I*_*h*_ is the mean intensity for reflection *h*. *R*_pim_ = Σ_*h*_(1/*n*_*h*_ − 1) Σ_*l*_ |*I*_*hl*_ − *I*_*h*_|/Σ_*h*_Σ_*l*_(*I*_*h*_). *R*_cryst_ = Σ‖*F*_o_| − |*F*_c_‖/Σ|*F*_o_|, where *F*_o_ and *F*_c_ are measured and calculated structure factors, respectively. *R*_free_ = Σ‖*F*_o_| − |*F*_c_‖/Σ|*F*_o_|, calculated from 5% of the reflections selected randomly and omitted during refinement

	Ndom389‐Aβ(4–10)	Ndom389‐Aβ(10–16)	Ndom389‐Aβ(1–16)	Ndom389‐Aβ(35–42)	Ndom389‐Aβ(4–10)Y
Resolution (Å)	1.90	1.80	1.80	1.55	1.65
Visible peptide	D7‐S8	E11‐V12/Q15‐K16	D7‐S8	I41‐A42	G9‐(NT)
Space group	*P1*				
Cell dimensions (Å; a, b, c); angle (°; α,β,γ) Molecule/AU	73.4, 101.8, 114.4; 85.2, 86.1, 81.4 4	73.3, 101.8, 113.9; 85.0, 85.6, 81.9 4	73.3, 101.7, 114.1; 85.1, 85.6, 81.3 4	73.0, 76.9, 83.2; 88.6, 64.1, 75.2 2	73.0, 76.5, 83.2; 88.8, 64.2, 75.6 2
Total/unique reflections	384,870/293,455	929,478/291,734	461,830/281,258	324,307/200,050	520,452/172,512
Completeness (%)	93 (84)^a^	97 (95.7)^a^	94 (82)^a^	88 (56)^a^	92 (64)^a^
*R*_merge_ a	7.1 (43.8)	12.0 (76.3)	6.1 (46.0)	4.2 (44.4)	8.1 (70.6)
*R*_pim_ a	7.1 (43.8)	7.9 (49.7)	6.0 (45.9)	4.2 (44.4)	5.4 (49.8)
*I*/σ(*I*)^a^	5.8 (1.4)	5.3 (1.4)	6.8 (1.4)	7.5 (1.4)	6.4 (1.3)
CC(1/2)	0.996 (0.816)	0.993 (0.325)	0.998 (0.635)	0.993 (0.565)	0.995 (0.318)
*R*_cryst_	18.5	19.7	18.0	15.8	20.8
*R*_free_	22.4	22.9	21.0	18.1	24.1
rmsd in bond lengths (Å)	0.016	0.012	0.011	0.014	0.011
rmsd in bond angles (°)	1.34	1.40	1.34	1.40	1.33
*B*‐factor statistics (Å^2^)					
Protein all atoms	27.4/24.8/22.2/23.1	29.3/26.3/24.3/27.0	26.5/23.3/21.2/23.4	33.0/37.8	27.9/31.0
Protein main chain atoms	26.4/23.7/21.1/22.3	28.4/25.2/23.4/26.2	25.2/22.1/20.0/22.3	30.8/35.4	26.7/29.9
Protein side chain atoms	28.4/25.8/23.2/23.9	30.2/27.3/25.2/27.9	27.7/24.6/22.4/24.5	35.2/40.1	29.1/31.1
Peptide atoms	26.0/23.5/24.6/23.3	27.5/23.5/21.9/28.1	27.4/27.3/23.5/26.3	28.4/28.9	41.7/44.6
Solvent atoms	29.7	31.0	30.9	44.8	35.3
Zn^2+^/Cl^−^ ions	16.7/16.2	19.2/19.7 (Na^+^ 33.5/Ca^2+^ 35.2)	17.5/16.7	25.5/26.9	19.9/21.5
Glycosylated carbohydrate atoms	48.6	54.1	47.7	67.3	58.9
Ramachandran statistics (molprobity)					
Favoured	98%	98%	98%	98%	98%
Outliers	0.2%	0.2%	0.2%	0.2%	0.2%
PDB code	5am8	5am9	5ama	5amb	5amc

a Values in parentheses refer to the highest resolution shell.

**Figure 6:**
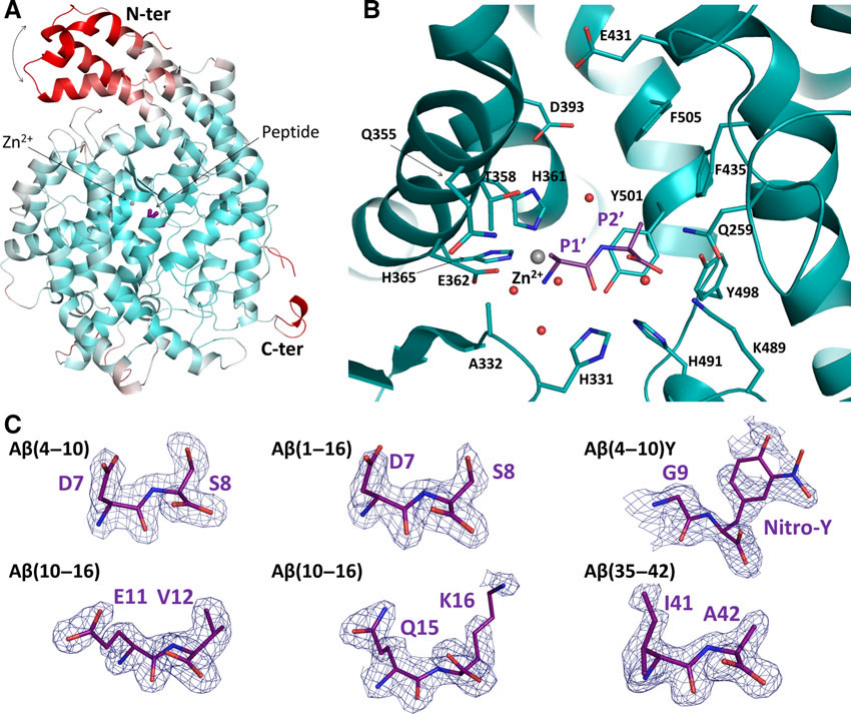
Structures, binding modes and interactions of Aβ peptide fragments with the N‐domain of ACE. (A) Overall structure of Ndom389 coloured in a *B*‐factor spectrum (white, low; red, high) to highlight the hinge region of the N‐terminal domain ‘capping’ the catalytic channel. The bound dipeptide is shown in purple, the catalytic zinc ion as a grey sphere. (B) Close‐up view of the catalytic site of the Ndom389 in complex with a bound dipeptide. The dipeptide, modelled as Ala‐Ala, reflects the common mechanism of binding observed in all complex structures with Aβ fragments. The dipeptide is shown in purple and the Ndom389 residues involved in binding are represented as sticks. The spheres represent the zinc ion (in grey) and water molecules (in red). (C) Portions of the Fourier electron density map at the site of the bound peptides. The map was generated using refmac5 74 and corresponds to the difference weighted 2*mF*_o_ − *DF*_c_ density map, contoured at 1.0σ level, in which the peptide atoms were omitted.

Overall, the structure of Ndom389 did not show any major conformational change upon peptide binding. The previously observed hinge motion of the N‐terminal helices 47 was slightly more pronounced in some of the molecules and resulted in a larger asymmetric unit (*a* = 73 Å, *b* = 102 Å, *c* = 114 Å; α = 85°, β = 86°, γ = 81°) with four Ndom389 chains, still in space group *P1*, for the Aβ(4–10), Aβ(10–16), Aβ(1–16) complexes (Table 6). The structures with Aβ(35–42) and Aβ(4–10)Y were in the same crystallographic cell as previously reported for Ndom389 with two chains per asymmetric unit in *P1* (*a* = 73 Å, *b* = 77 Å, *c* = 83 Å; α = 89°, β = 64°, γ = 75°). The degree of movement appeared limited and could not be correlated to the size of the substrates or bound peptides, but caused visible disorder in the N‐terminal region of Ndom389 and is highlighted by higher *B* factors (Fig. 6A).

In each of the structures, electron density was clearly observed in the S′ side of the Ndom389 catalytic pocket for what corresponds to a dipeptide. Electron density maps for the side chains of sites P1′ and P2′ of the peptides were interpreted as the products of the prolonged reactions of Aβ cleavage by Ndom389 (Fig. 6C). The carboxy‐dipeptide residues of Aβ(35–42) and Aβ(4–10)Y were visible, Ile41‐Ala42 and Gly9‐3‐nitrotyrosine (NT) 10 respectively. Both Aβ(4–10) and Aβ(1–16) presented the Asp7‐Ser8 residues. The structure of Ndom389 with Aβ(10–16) was unique in offering two alternative dipeptides, each present in two of the chains forming the asymmetric unit, and corresponding to Glu11‐Val12 and Gln15‐Lys16. Coincidentally, the Aβ(10–16) structure also showed unusual ion coordination in proximity to the binding site. In the presence of Glu11‐Val12 a cation is octahedrally coordinated by Glu262, Asn263 and Asp354 of Ndom389 along with three water molecules, while the same residues are involved in cationic interaction with only two water molecules when Gln15‐Lys16 is bound. The ion coordination was carefully analysed in each case using the cmm validation server 50 and interpreted as calcium and sodium ions, respectively. Both are present in the crystallization conditions but are unlikely to have any physiological role and have not been observed in any of the other N‐domain crystal structures.

### Mode of peptide binding in N‐domain S′ pockets

**Figure 7:**
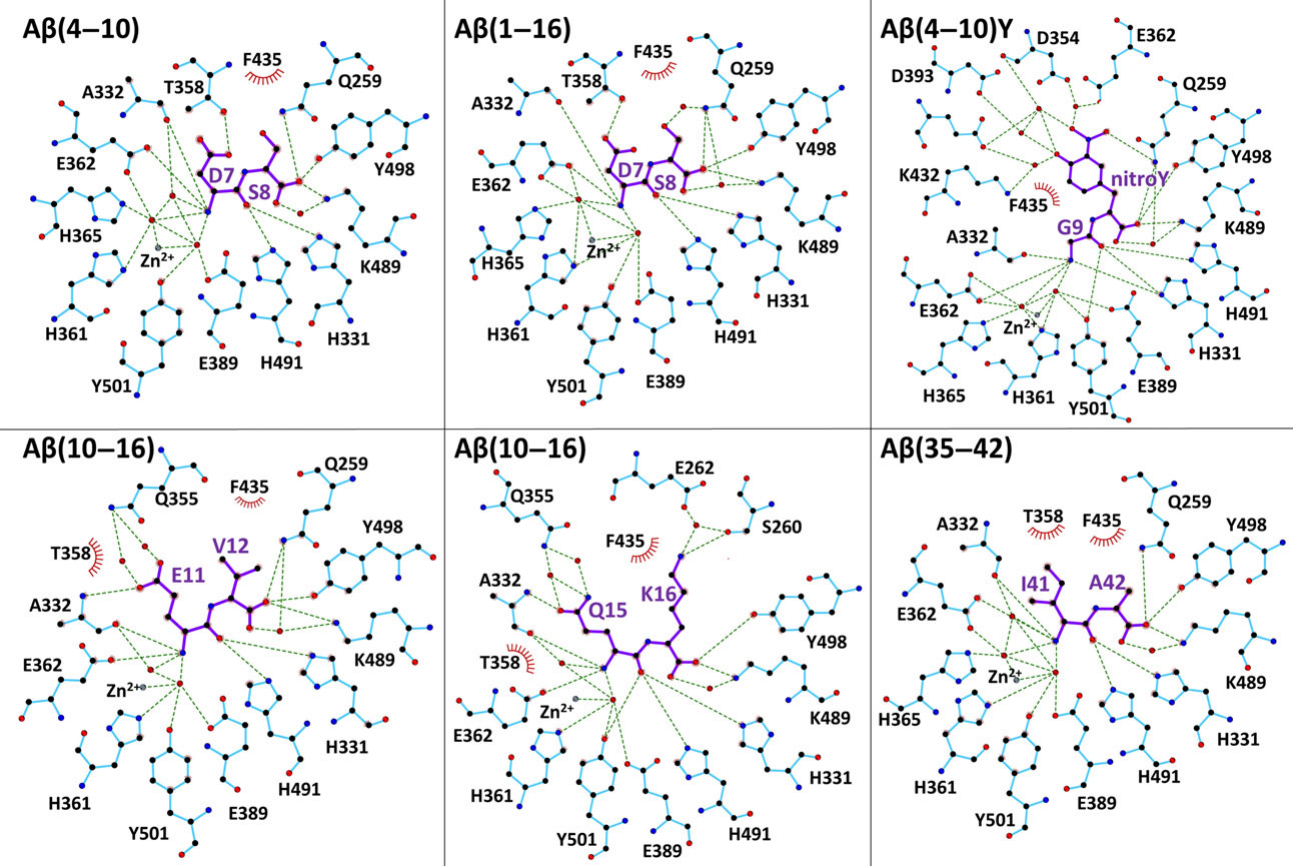
Mechanism of Aβ fragment binding to the N‐domain. Schematics of peptide binding to the N‐domain. Interactions were calculated with ligplot ^+^ 77. The two alternative peptides for Aβ(10–16) are included with Glu11‐Val12 and Glu15‐Lys16 observed in chains A, D and B, C respectively.

The crystal structures of Ndom389 in complex with the Aβ fragments present a common mechanism of peptide binding within the S′ catalytic pocket (Fig. 6B). The Ndom389 essentially recognizes the main chain of the peptides through seven hydrogen bonds. The P1′ position interacts with the main chain of Ala332 and the side chains of Glu362, His491 and His331. The S2′ pocket is composed of Gln259, Lys489 and Tyr498 whose polar side chains anchor the carboxy‐terminal end of the peptide. Furthermore, a network of water molecules was also observed in all structures that further mediates interaction of the peptides’ main chain with residues Ala332, Glu362, Tyr501 and Lys489 of Ndom389 (Fig. 7). The N‐terminal groups of the peptides are held in close proximity to the zinc ion via two coordinating water molecules.

Additional and more specific contacts were observed with peptides Asp7‐Ser8 where the side chain of Thr358 makes a hydrogen bond with the acidic group of Asp7, and a water‐mediated bond exists between Gln259 and the Ser8 side chain (Fig. 7). Interestingly the two alternative peptides seen within the structure of Aβ(10–16) show similar contacts at the P1′ position with the longer side chain of Glu11 or Gln15 able to make direct contact with Ala332 and two water‐mediated interactions with Gln355 (Fig. 7). The side chain of residues at P2′ may be held in position by the surrounding hydrophobic residues Phe435, Tyr501 and Phe505. Additionally, the Lys16 ε‐amino group is within distance (5.5 Å) of a potential cation–π interaction with Phe435. In the case of Aβ(4–10)Y, the larger nitrotyrosine fits well within the S2′ pocket. The additional nitro group is within hydrogen‐bond distance of Gln259 and the hydroxyl group can make water‐mediated interactions further down the catalytic channel with residues Asp393 and Glu431. Electron density was observed to be weaker in both molecules of the asymmetric unit for the nitrotyrosine which may be indicative of some flexibility (Fig. 7).

## Discussion

In order to understand the potential differences between the two domains of ACE and their synergy in full‐length sACE we have investigated the metabolism of the Aβ peptide by single and double domain recombinant ACE forms. Previous studies have noted potential discrepancies in kinetic data and a lack of substrate specificity based on different sources and forms of ACE 46, 51. Thus, all of the constructs were purified from one cell line of Chinese hamster ovary (CHO) cells and assay conditions were as physiological as possible.

The Aβ(1–16) peptide was cleaved at the His14‐Gln15 bond by all the ACE constructs in our kinetic experiments, in contrast to the full‐length Aβ(1–42) where multiple cleavage sites have been identified for ACE 20, 22, 23, 24, 25, 26. The Aβ(1–16) cleavage site at His14‐Gln15 is typical of ACE's exoprotease action, cleaving the penultimate C‐terminal peptide bond. This type of cleavage has been observed in previous studies with other amyloid peptides 23, 24 and was also observed in the crystal structures of the N‐domain in complex with all the Aβ fragments tested. Based on the MS analysis of this study (Tables 4 and 5), the primary cleavage of Aβ(1–16) occurs between residues His14 and Gln15 and was also observed in the Ndom‐Gln15‐Lys16 crystal structure of Aβ(10–16) (Fig. 6C).

Surprisingly the crystal structure of N‐domain with Aβ(10–16) also presented an alternative cleavage site at Tyr10‐Glu11, which is probably the result of successive dipeptidyl cleavages of the Aβ fragment by the N‐domain during crystal growth. Further degradation was also observed with Aβ(4–10) and Aβ(1–16) which actually presented residues Asp7‐Ser8 in the structures, indicative of a His6‐Asp7 cleavage site. In contrast to the suggested crystal structure cleavage site, consecutive cleavage sites of Aβ and cleavage of the Asp7‐Ser8 site, Aβ(1–7) product, were found after prolonged incubation of Aβ(1–16) with both N‐ and C‐domain forms of ACE (Table 5). However, Aβ(1–16) hydrolysis at His6‐Asp7 was only seen after a 24 h incubation with C‐sACE and no N‐domain constructs generated this hydrolysis product (Table 5). It is plausible that the N‐domain constructs degraded it faster after 24 h and thus it was not detected. Combined, the MS analysis and crystal structures of the peptides generated by digestion of Aβ(1–16) with Ndom showed that it undergoes both endoproteolytic and exoproteolytic cleavage with prolonged incubation (Table 5).

Fluorogenic ACE substrates have been widely used to investigate the mechanism of substrate processing and inhibitor binding 7, 45, 52. The kinetic assays indicated that both fluorogenic peptides were endoproteolytically cleaved at the previously identified Asp7‐Ser8 bond 20. Again, the cleavage site was consistent across all ACE variants. This is in contrast with the crystal structure of the Ndom389–Aβ(4–10)Y complex that presented Gly9‐NT10, the expected product of the dipeptidylpeptidase activity. Interestingly, previous assays on ACE fluorescence resonance energy transfer substrates indicate that the large C‐terminal ethylenediamine 2,4‐dinitrophenyl (EDDnp) groups usually occupy the S2′ pocket and cleavage would take place one residue away from this acceptor group 7, in accordance with the crystal structure. In our assays, cleavage was consistently three residues from the EDDnp and NT groups which are relatively similar in charge and size. These results offer evidence that the fluorogenic peptide may thus be subjected to either endoproteolytic activity or classical dipeptidylpeptidase activity depending on the environmental conditions. Further work is required to understand the extent of ACE's endoproteolytic capabilities.

Both biochemical and structural data show the ability of ACE to cleave peptides of diverse length and composition. Although the crystal structures with N‐domain only presented dipeptide fragments, this has facilitated our understanding of the enzyme's broad catalytic activity. This is explained by a common mode of peptide binding to N‐domain, which principally targets the C‐terminal P2′ position to the S2′ pocket and recognizes the main chain of the P1′ peptide. This mechanism is reminiscent of C‐domain binding to angiotensin II and the bradykinin potentiating peptide b (BPPb) 53. The residues involved in binding within the S′ pockets are conserved in both ACE domains. The N‐domain selectivity for Aβ is therefore most probably conferred through interactions with the non‐prime binding site 47, 53. Additionally, the N‐domain should be able to accommodate larger substrates through movement of the N‐terminal helices, as suggested by the disorder of the hinge region in the crystal structures and in parallel with the observed ‘open’ conformation of C‐domain when bound to BPPb. Although the chloride ion is expected to play a role in substrate binding and domain selectivity 54, the structures presented here did not provide any further evidence of this.

In addition to the putative shared mode of peptide recognition, the multiple fragments, observed across all high resolution structures, displayed some common interactions. Two groups can be distinguished at P1′ where peptides with longer polar side chains (i.e. Asp7, Glu11 and Gln15) can make hydrogen bonds with Ala332 or Thr358 of Ndom389. Noticeably, Thr358 is a residue unique to the N‐domain found at the entrance of S2′ pocket and may drive selectivity to a degree. Second, hydrophobic residues, such as Ile41, at the P1′ position of the substrate have also been shown to be better cleaved by both domains of ACE 7. The S1′ and S2′ pocket offers strong hydrophobic interactions for the dipeptides Glu11‐Val12, Ile41‐Ala42 and the Gly9‐NT10 of Aβ(1–16), Aβ(1–42) and Aβ(4–10)Y, respectively. Furthermore, a longer polar side chain may also be stabilized by electrostatic interactions (e.g. Lys16) deeper in the S2′ pocket as well as through a network of water molecules as with the nitrotyrosine group of Aβ(4–10)Y.

Interestingly, N‐domain selectivity is affected in the full‐length forms of ACE across all substrates. That is, the sACE with inactivated C‐domain has lower *k*_cat_ values for both Aβ(1–16) and Aβ(4–10)Q than N‐domain. This decrease in turnover rate substantially reduces the N‐domain selectivity of the full‐length knockouts. The sACE form appears to improve the interaction of the Aβ(4–10)Y with the N‐domain of N‐sACE and slow the turnover rate. The opposite is true for Aβ(4–10)Q.

Despite a general decrease in selectivity of the N‐sACE, our results indicate that amyloid peptides are far more N‐domain selective in the current optimal C‐domain conditions (higher NaCl concentrations). However, in cells expressing Aβ there was no overall difference in the hydrolysis of Aβ between C‐sACE, N‐sACE and sACE 22. This is probably due to the fact that the C‐domain of sACE might only hydrolyse Aβ effectively when the full‐length protein is anchored in the cell membrane 22.

The two active sites within human and bovine sACE exhibit negative cooperativity with synthetic tripeptide substrates 44, 45, 55. Further work with physiological substrates and human ACE enzymes showed a similar result for angiotensin I and angiotensin‐(1‐7) but not angiotensin‐(1‐9), implying that different substrates result in varying synergistic effects of the two domains 46. Similar to other kinetic observations with physiological substrates, such as angiotensin‐(1‐9) and bradykinin, the degree of cooperativity between domains is considerably less than many synthetic peptides observed previously 46, 56. The sACE CC‐domain enzyme possessed a catalytic ability per active site similar to the individual domains, indicating an additive effect between the two C‐domains with Aβ(4–10)Y substrate.

Given the discrepancy between the magnitude of selectivity of the truncated domains and the sACE forms, there are most certainly cooperative effects taking place with regard to the Aβ substrate. The equivalent affinity of the truncated and full‐length constructs to Aβ(1–16) and the largely varying turnover rates suggest a close interaction between the two domains and the binding and hydrolysis of their individual active sites. The cooperative effects of the two domains towards Aβ(1–16), overall, are negative. Due to the C‐domain's poorer hydrolysis of Aβ we can infer that the C‐domain appears to negatively regulate the N‐domain in sACE. This is also true for the Aβ(4–10)Q substrate, although to a much lesser extent. There is a large shift in both level of cooperativity and type of cooperativity [see Aβ(4–10)Y below] on comparing the truncated domains to the full‐length knockouts. For instance, the magnitude of the Aβ(1–16) negative cooperative effect decreases on comparing the truncated domains to the sACE constructs, whereas Aβ(4–10)Q has no shift. The positive effect of the N‐domain can be illustrated through C‐sACE. Here, C‐sACE exhibited improved activity compared to the truncated Cdom for all Aβ substrates, suggesting that the N‐domain is critical for this synergistic effect to occur. This notion is further supported by the poor activity of the CC‐sACE and its interaction with both Aβ(1–16) and Aβ(4–10)Q, confirming that the mere presence of another domain did not improve the C‐domain hydrolysis of Aβ peptides. Previously, we showed that the presence of the N‐domain had regulatory effects on hydrolysis of substrates Cbz‐Phe‐His‐Leu and (Abz)‐LFK(Dnp)‐OH 55. Taken together, these data suggest that the effects of domain interactions could be influenced by the type of substrate.

The cooperativity in the sACE molecules, however, was altered by the presence of Aβ capping groups. When one takes the truncated domains into account there is a negative effect on the activity of sACE. On examination of the N‐sACE and C‐sACE constructs, a positive form of cooperativity is now found towards Aβ(4–10)Y. The two catalytically active domains, binding and hydrolysis, are affected by both the structural arrangement and the activity of their respective active sites. This could be ascribed to the fact that EDDnp quenchers require attachment of a Gln or Glu residue, both of which could alter selectivity and interdomain cooperativity due to the length and polarity of their side chains.

Based on our results and the synergistic effect that the C‐domain exerts on the N‐domain, the C‐domain activity is likely to be important *in vivo*. Oba *et al*. 21 discovered that the larger Aβ(1–40) substrate did not out‐compete smaller substrates like hippuryl‐l‐histidyl‐ l‐leucine, suggesting that the structure of the C‐domain was not conducive to binding of large Aβ peptides. This observation is supported by the architecture of the central cavity found in tACE (human testis ACE, C‐domain equivalent) crystal structure and the chloride‐dependent substrate interactions 54, 57. The latter study, however, was performed on recombinant truncated domains only. It is also important to note that Aβ occurs in many forms. In this study we examined the physiological substrate Aβ(1–16), which is over‐secreted in AD 42, contains the metal binding domain 41, 58, forms soluble dimers 59 and may have metal associated toxic effects 38, 59, 60. Smaller Aβ molecules, like Aβ(1–16), may have better access to the C‐domain. Indeed, the C‐domain of sACE most certainly has some Aβ affinity, as is evident from the mass spectrometry and kinetic data on Aβ(1–16) and the fluorescence resonance energy transfer Aβ(4–10)Q and Aβ(4–10)Y peptides. One might speculate that the structure of the C‐domain in sACE is more open due to the physical, and specific, presence of the N‐domain. The N‐domain, possibly, stretches out the flexible lid of helices α1–α3 in the C‐domain making substrate access more efficient 61. Fluorogenic peptides may not necessarily be the best at indicating selectivity as the addition of artificial groups on either end bias domain specificity based on their size, charge and additional amino acids required to attach them 7, 62. It is also possible for these groups to stabilize an interaction that would not necessarily take place 62 such as the ones observed in the crystal structure where the nitrotyrosine group of Aβ(4–10)Y offers a number of additional interactions.

In summary, we have assessed the domain selectivity and cooperativity of Aβ peptide hydrolysis. This further emphasizes the dynamic roles of the two domains of sACE in substrate processing. This dynamism is not conserved across all ACE substrates, but rather appears dependent on the nature of the substrate itself. Our study provides further evidence of both endoproteolytic and classical dicarboxypeptidase activities of ACE on Aβ peptides. The crystal structures presented also offer a molecular framework to better understand peptide binding to the N‐domain.

## Experimental procedures

### Enzymes

For single, soluble enzymatic domains, a modified tACE construct, tACEΔ36NJ, that lacks the transmembrane region and unique 36 amino acid N terminus (and is therefore identical to the sACE C‐domain; referred to as Cdom) had been generated previously 63. A soluble form of the N‐domain, consisting of amino acids 1–629 of sACE (referred to as Ndom), in vector pECE was obtained from S. Danilov (University of Illinois at Chicago, IL, USA) and was cloned into sequencing vector pBlueScript SK+ (Invitrogen, Carlsbad, CA, USA) as previously described 61, 64. For all crystallization experiments a minimally glycosylated form of the soluble N‐domain (Ndom389) was expressed and purified as previously described 47.

The full‐length domain knockouts of sACE, both in pECE, were obtained from V. Dive (CEA, France) and were constructed by Wei *et al*. 5 through site directed mutagenesis 65. For the construction of the N‐sACE, the His361 (CAT) and His365 (CAT) were converted to Lys (AAG and AAA respectively). Similarly C‐sACE was generated by mutating sites His959 (CAC) and His963 (CAC) to Lys (AAA and AAG respectively). Both constructs have the complete signal, transmembrane and stalk region corresponding to full‐length sACE. The CC‐sACE was constructed in our group as previously described 55; briefly, it consists of two C‐domains joined by the sACE interdomain linker region as well as the juxtamembrane stalk and transmembrane region in the mammalian expression vector pLEN (Metabolic Biosystems, Mountain View, CA, USA) (Fig. 1).

### Expression and purification of enzymes

All enzymes were expressed in CHO cells using standard tissue culture approaches as formerly described 66. All enzymes were purified using lisinopril‐sepharose affinity chromatography as previously described 67 with the following considerations: single domains were isolated from the harvest medium while full‐length enzymes were purified from whole cell triton lysates. N‐domain constructs required the addition of 800 mm NaCl to medium/lysates for effective purification 68. ACE activity was detected using the substrate Cbz‐Phe‐His‐Leu 69 and pooled enzyme was dialysed twice with 2 L of 5 mm Hepes (pH 7.5). All enzymes were concentrated and stored at 4 °C in 50 mm Hepes (pH 7.5). Enzyme integrity and purity were assessed by SDS/PAGE and subsequent Coomassie staining.

In order to determine loss of enzymatic activity due to storage, specific activities were calculated immediately after purification. It was assumed that enzyme which is eluted off the lisinopril column must be active in order to bind the ligand. Specific activities were then redetermined prior to kinetic analysis and protein concentrations adjusted accordingly.

### Amyloid kinetics

ACE and Aβ(1–16) (H‐DAEFRHDSGYEVHHQK‐OH) (Bachem AG) hydrolysis assays were performed in reaction tubes and transferred to HPLC vials for analysis using the Agilent 1260 Infinity HPLC. Mixtures were separated on a Poroshell 120 EC‐18 column with a 2.7 μm pore size. The reaction consists of 25 μL substrate incubated with 25 μL enzyme for 15 min at 37 °C and was stopped with the addition of 10 μL of 0.25% trifluoroacetic acid (TFA). The total reaction (60 μL) was cleared of contaminants on a spin column (GHP Nanosep^®^ MF Centrifugal Device 0.45 μm pore size) and run on the HPLC. Kinetic parameters were determined through the fitting of initial rates to the Michaelis–Menten equation with graphpad prism software (v 4.01, GraphPad Prism^®^).

Fluorogenic substrates designed around the established N‐terminal Asp7‐Ser8 cleavage site of the full‐length Aβ(1–42) were used to develop a higher throughput assay. There are two variations of the short fluorogenic peptides: Aβ(4–10)Q (Abz‐FRHDSG(Q)‐EDDnp) which has an N‐terminal Abz donor and a C‐terminal EDDnp quencher group attached to an additional Gln residue. The second peptide, Aβ(4–10)Y (Abz‐FRHDSG‐(NT)) has a different quencher molecule, NT, which substitutes for the naturally occurring Tyr residue.

### Determination of cleavage site

Kinetic reaction digests, 24 h digests and undigested Aβ(1–16) samples were collected off the HPLC, dried down with the Savant SpeedyVac (ThermoScientific, USA) and then resuspended in 50% acetonitrile (ACN) prior to being sent for mass spectrometry. The Aβ(4–10)Q and Aβ(4–10)Y digested and undigested samples were cleaned over a C18 Zip Tip^®^ (Millipore) and sent for mass spectrometry. The mass spectrometry was analysed at the Centre for Proteomic and Genomic Research (Cape Town, South Africa). Briefly, the collected peptides were spotted onto a 10 mg·mL^−1^ α‐cyano‐4‐hydroxycinnamic acid matrix (Fluka, USA) in 80% ACN, 0.2% TFA for a final concentration of 5 mg·mL^−1^ matrix in 40% ACN, 0.1% TFA, 10 mm NH_4_H_2_PO_4_. Mass spectrometry was performed with a 4800 MALDI‐TOF/TOF (Applied Biosytems) with all spectra recorded in positive reflector mode. Spectra were generated with 400 laser shots/spectrum at a laser intensity of 3800 (arbitrary units) with a grid voltage of 16 kV.

### Determination of kinetic parameters

#### Aβ(1–16)

Enzyme reactions were initiated on the addition of 25 μL enzyme (at a final concentration within 10% hydrolysis of total substrate; see Table 1) to 25 μL Aβ(1–16) in Hepes buffer (50 mm Hepes pH 7.5, 100 mm NaCl, 10 μm ZnSO_4_ buffer), ranging in concentration from 0 to 45 μm. The reaction was incubated for 15 min at 37 °C and stopped with the addition of 10 μL 0.25% TFA to make up a total volume of 60 μL. The reactions were performed in triplicate and 50 μL injections were analysed via HPLC (Agilent Technologies) across a gradient of 0.1% TFA and 2% ACN in water to 0.1% TFA, 95% ACN. A calibration curve was set up to convert the product peak area to picomoles product formed, through the complete hydrolysis of the substrate with the N‐domain. The initial rates of reactions were generated by converting and plotting the resultant peak product area, and were used to assess enzyme activity. Kinetic constants were calculated using the Michaelis–Menten method using graphpad prism software (v 4.01, GraphPad Prism^®^).

#### Aβ(4–10)Q

Hydrolysis of the fluorogenic peptide Abz‐FRHDSG(Q)‐EDDnp (Aβ(4–10)Q, obtained from A. Carmona, Universidade Federal de São Paulo, Brazil) was performed in Hepes buffer (50 mm Hepes pH 7.5, 100 mm NaCl, 10 μm ZnSO_4_ buffer) with 150 μL enzyme (at final concentrations within 10% hydrolysis of total substrate; see Table 2) and equal volume of substrate ranging from 0 to 30 μm. The assay is a modified form of the continuous assay 7 where the assay is set up on ice in triplicate in a 96‐well plate. The baseline fluorescence was at time zero and then the substrate was incubated at 37 °C and fluorescence read at the 45 min time point on a Cary Eclipse spectrofluorimeter (Varian Inc.) at λ_ex_ = 320 nm and λ_em_ = 420 nm. Again, kinetic constants were calculated using the Michaelis–Menten method with graphpad prism software (v4.01, GraphPad Prism^®^). The detected fluorescence was converted to picamoles product formed through the slope of a calibration curve of Abz‐Gly. A correction curve was also applied to correct the data for the inner filter effect 70.

#### Aβ(4–10)Y

An alternative fluorogenic peptide, Abz‐FRHDSG‐(NT) (BIOPEP^™^ South Africa) was assayed, with 100 μL enzyme and 100 μL substrate (at a final enzyme concentration within 10% hydrolysis of total substrate; see Table 3) as above, in 96‐well plates. The samples were read at the 5 min time point on a Cary Eclipse spectrofluorimeter (Varian Inc.) at λ_ex_ = 320 nm and λ_em_ = 420 nm. Kinetic constants, inner filter effect and standard curves were calculated as above.

### X‐ray crystallography

The crystals of Ndom389 in complex with Aβ peptides were obtained by co‐crystallization with a 2.5 mm peptide. Peptides Aβ(1–16) (Sigma, SCP0052), Aβ(10–16) (Sigma, SCP0031), Aβ(4–10) (GenScript, RP20173), Aβ(35–42) (GenScript RP20145) and Aβ(4–10)Y were used. Crystals were grown with 1 μL of the peptide (5–10 mg·mL^−1^ in 50 mm Hepes, pH 7.5, 0.1 mm PMSF) mixed with an equal volume of reservoir solution consisting of 30% PEG 550 MME/PEG 20000, 100 mm Tris/Bicine, pH 8.5, and 0.06 m divalent cations (Molecular Dimensions) and suspended above the well as a hanging drop. Crystals of better quality were obtained after two to three cycles of macro‐seeding.

X‐ray diffraction data were collected on station IO3 at the Diamond Light Source (Didcot, UK). Crystals were kept at constant temperature (100 K) under the liquid nitrogen jet during data collection. Images were collected using a PILATUS‐6M detector (Dectris, Switzerland). Raw data images were processed and scaled with mosflm 71 and scala using the ccp4 suite 6.5 72. Initial phases for structure solution were obtained using the molecular replacement routines of the phaser program 73. The atomic coordinates of N‐domain (PDB code 3NXQ 47) were used as a search model for structure determination. The resultant models were refined using refmac5 74. Manual adjustments of the model were carried out using coot 75. Water molecules were added at positions where *F*_o_ − *F*_c_ Fourier difference electron density peaks exceeded 3σ and potential hydrogen bonds could be made. Validation was conducted with the aid of the program molprobity 76. Crystallographic data statistics are summarized in Table 6. All figures were drawn with pymol (Schrödinger, LLC, New York, USA). Hydrogen bonds were verified with the program ligplot ^+^ 77.

### Statistical analysis

Statistical analysis of the data was determined by Student's *t* test. Differences with *P* < 0.05 were considered statistically significant. Fractional error was calculated for the *k*_cat_/*K*_m_ ratio with the following equation:

$$\Delta Z=Z\sqrt{{\left(\frac{\Delta a}{a}\right)}^{2}+{\left(\frac{\Delta b}{b}\right)}^{2}}$$

where *Z* represents the mean *k*_cat_/*K*_m_ and Δ*Z* represents the fractional error. The Δ*a* represents the SEM of the mean *k*_cat_ (*a*). Similarly, Δ*b* represents the SEM of the mean *K*_m_ (*b*).

## Conflict of interest

The authors have no conflict of interest to declare.

## Author contributions

KL contributed to the conceptualization of the project, carried out the MS and kinetic analyses and wrote the paper. GM performed all the structural biology experiments, analysed the data and wrote a major part of the paper. RGD contributed to critical discussion of the results and to writing the paper. SLS contributed to the purification of the ACE constructs and the MS analysis. EDS conceived the project, analysed the data and wrote the paper. KRA conceived the structural biology part of the experiments, analysed the data and edited the paper. All authors reviewed the results and approved the final version of the manuscript.
